# Multimodal targeting of glioma with functionalized nanoparticles

**DOI:** 10.1186/s12935-022-02687-8

**Published:** 2022-08-23

**Authors:** Hany E. Marei

**Affiliations:** Department of Cytology and Histology, Faculty of Veterinary Medicine, Mansoura University, Mansoura, 35116 Egypt

**Keywords:** Glioma, Nanoparticles, Chemotherapy, Radiotherapy, Immunotherapy, Immune checkpoint modulators, CAR T cells, Vaccine-based immunotherapy

## Abstract

The most common and aggressive primitive intracranial tumor of the central nervous system is the glioma. The blood–brain barrier (BBB) has proven to be a significant obstacle to the effective treatment of glioma. To effectively treat glioma, different ways have been used to cross the BBB to deliver drugs to the brain. Drug delivery through nanocarriers proves to be an effective and non-invasive technique for the treatment of glioma and has great potential in the treatment of glioma. In this review, we will provide an overview of nanocarrier-mediated drug delivery and related glioma therapy. Nanocarrier-mediated drug delivery techniques to cross the BBB (liposomes, micelles, inorganic systems, polymeric nanoparticles, nanogel system, and biomimetic nanoparticles) are explored. Finally, the use of nanotherapeutic approaches in the treatment of glioblastoma including chemotherapy, radiotherapy, photothermal therapy, gene therapy, glioma genome editing, immunotherapy, chimeric antigen receptor (CAR) T-cells, immune checkpoint modulators, immune photothermal therapy, vaccine-based immunotherapy**,** and combination therapy is summarized. Furthermore, this article offers various views on the clinical applicability of nanomedicine.

**Supplementary Information:**

The online version contains supplementary material available at 10.1186/s12935-022-02687-8.

## Background

Gliomas are types of primary brain tumours that have a poor prognosis due to their high invasive potential and aggressive clinical course. They account for over 80% of all malignant primary brain malignancies and 30% of all brain tumours [1]. Glioblastoma multiforme (GBM) is a type IV astrocytoma that affects adults. It has an annual global incidence of 3.22 per 100,000, accounting for 54.7 percent of all gliomas and 16 percent of all primary brain and central nervous system malignancies [2, 3]. Integration of molecular and histological features based on the most recent World Health Organization (WHO) classifications has enhanced not only glioma diagnosis and prognosis, but also the prospect for precision targeting based on patient’s specific molecular patterns [4, 5]. Gliomas are classified into four grades (I to IV) by the WHO in 2016, and patients with grade-IV gliomas have an average survival duration of only 15 months [4, 5]. Despite the fact that gliomas have a multimodal therapeutic regimen that includes maximum surgical resection followed by concurrent radiation or chemotherapy, treatment is frequently insufficient to stop tumour development [6].

The current treatment of GBM includes a complete surgical excision of the tumour mass, followed by radiation and temozolomide chemotherapy [7], it only ensures a median survival of 15 months. The blood brain barrier (BBB)’s separative function, the high heterogeneity of glioma cells, and the hostile inhibitory tumour microenvironment (TME) are among the main factors that not only promote glioma progression, but also reduce the therapeutic efficacy of standard surgical and radio-chemical therapeutic modalities [8, 9].

One of the drawbacks of intravenous chemotherapy treatment is the high percentage that does not reach the brain, as well as the ensuing side effects that result from accumulation in non-target organs. This, in turn, limits dose and reduces quality of life by causing damage to peripheral organs such the heart, lungs, and liver [10]. It is critical that safer, more effective, and efficient targeted treatments for patients with brain cancer be developed, and nanomedicine offers potential solutions to this challenge.

The therapeutic potential of nanoparticle (NP)-based brain-targeting drug delivery systems has yet to be fully realized, owing to the fact that the majority of them are lost during the delivery process. The general strategies for glioma targeting delivery of a systemically administered NPs-based brain-targeting drug delivery system include a six-step CRITID delivery cascade: circulation in systemic blood, receptor recognition on the blood–brain barrier (BBB), intracellular transport, diseased cell targeting after entering parenchyma, internalization by diseased cells, and finally intracellular drug release. Before discussing the use of nanoparticles in various glioma treatment modes, interested readers should review the general strategies for glioma targeting delivery, which have been summarized in several reviews [11–13].

The glioma targeting can be achieved either passively through the EPR effect or actively through the addition of targeting moieties to the surface of the nanoparticles. These two mechanisms have been discussed extensively elsewhere [14, 15]. However, people realize that the EPR effect is highly heterogeneous and does not always hold up in clinical settings and the magnitude of the EPR effect as seen in rodent models fails to translate to the clinic. The debate here is whether BBB is still intact in some of the tumor regions that glioma cells can hide. If yes, how can we achieve targeting these tumor cells using tailored nanoparticles? If no, how efficiently nanoparticles can passively accumulate in the glioma through leaky vessels? Some papers reported that EPR in glioma was much week than other types of cancers due to the presence of BBTB [16]. The tumor targeting efficiency depends on the size of nanoparticles. As for the active targeting, there are also some interesting findings regarding the protein corona [17, 18]. Previously, the active targeting was thought to be realized solely by attached ligands and their receptors. However, the complex proteins adsorbed on the surface of nanoparticles would be a crucial factor for in vivo tropism of these nanoparticles [19]. The presence of a corona on a NP surface may also mask the targeting ligands, which might not be able to bind the corresponding receptor. For example, transferrin-functionalized nanoparticles lost their targeting capabilities when a biomolecule corona adsorbed on the surface [20].

**Fig. 1:**
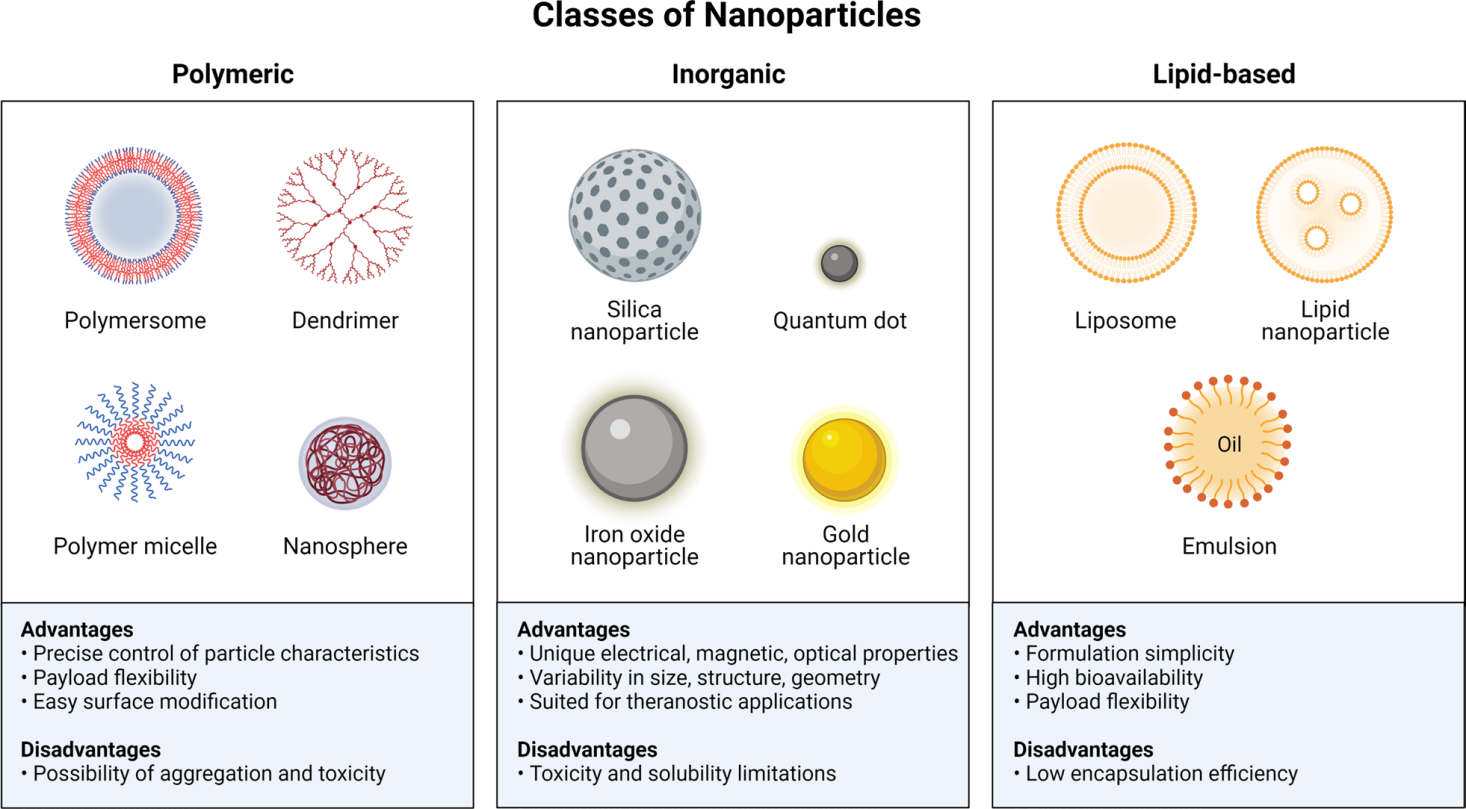
Advantages and disadvantages of different classes of nanoparticles

Polymeric nanoparticles (PN) [21, 22], dendrimers [23], liposomes [24], and solid lipid nanoparticles (SLN) [25] have all been developed as drug delivery systems to improve the transport of currently available antitumoral agents for glioblastoma multiforme (GBM). The different classes of nanoparticles used for the treatment of glioma including their advantages and disadvantages are summarized in (Fig. 1). Nanomedicine has several advantages over traditional cancer therapies, including multifunctionality, effective drug transport, and regulated drug cargo release [26]. In nanomedicine, efficient drug delivery can be achieved either passively through the increased permeability and retention (EPR) effect or actively through the addition of targeting moieties to the surface of the NPs [27, 28]. pH, heat, and enzyme induced release are examples of controlled release techniques in nanomedicine [29]. Nanostructured systems can help treat GBM by delivering medications across the BBB, promoting precise tumour cell targeting, increasing drug bioavailability, controlling drug release rates, protecting drugs from enzymatic degradation, and reducing systemic side effects [21, 24, 25, 30].

In this review, the primary targeting ligands utilized in nanosystems to circumvent the BBB and promote active targeting of medicines for glioblastoma are discussed. The benefits of utilizing functionalized nanoparticles in the treatment of glioma are also explored.

## Nanomedicine for glioma

Nanosystems are nanostructures that range in size from 1 to 1000 nm. However, it is well known that systems with sizes between 20 and 200 nm have better biological characteristics, such as greater internalization in tumour cells and the ability to bypass the BBB via two main endocytosis routes: clathrin- and caveolin-mediated endocytosis [31, 32]. The features of nanoparticles, such as particle size, shape, surface charge, and composition, are largely related to their physicochemical qualities [33]. The passive targeting mechanism known as the increased permeability and retention (EPR) effect allows nanosystems to concentrate in solid tumour tissues due to their physicochemical properties. Nanosystems can easily permeate through fenestrated arteries created during the angiogenesis process and accumulate in tumour tissue due to the weak lymphatic drainage system around the tumour, according to the EPR effect theory. Nanosystems may thus be able to enter tumour tissue in the latter stages of GBM. Surface charge is another significant physicochemical feature that can affect nanosystems’ in vivo function. Positively charged nanoparticles interact with biological membranes more effectively than neutral and negative nanoparticles. The electrostatic interaction between positively charged nanoparticles and the negative surface charge of BBB endothelial cells causes nanoparticle internalization via adsorptive-mediated endocytosis, which culminates in nanoparticle internalization. Positively charged nanoparticles, on the other hand, cause the generation of reactive oxygen species (ROS), increasing their toxicity and limiting their utilization through invasive routes [33].

Because these nanosystems have a limited sensitivity for tumoral cells, they can spread throughout the brain, causing injury to normal tissues [34]. To circumvent this constraint, the nanosystem surfaces have been modified with targeting ligands that can be recognized selectively by specific or overexpressed tumoral cell receptors (e.g., folate, transferrin, neurokinin-1, and v3 integrin receptors), a delivery strategy known as active targeting [35]. Additionally, several of these ligands can be recognized by BBB receptors, enhancing the nanosystem’s potential to cross the BBB via receptor-mediated transcytosis (e.g., transferrin receptor, albumin transporters, glucose transporter 1 (GLUT1), lactoferrin receptor, and folate receptor) [32]. Several targeting ligands are used to promote active targeting of nanosystems to gliomas cells including proteins [36], peptides [37], aptamers [38].

Diagnostic, therapeutic (radiation dose enhancers, hyperthermia inducers, drug delivery vehicles, vaccine adjuvants, photosensitizers, and enhancers of immunotherapy) and theranostic (combining both diagnostic and therapeutic) applications of metal nanoparticles have all been widely used in clinical practice [39]. One of the promising agents is gold nanoparticles (AuNPs). They have a number of advantages, including biocompatibility, well-established methods for synthesis in a wide range of sizes, and the ability to coat the surface with a variety of compounds to provide surface charge or interactivity with serum proteins [39]. A list of currently ongoing clinical trials using nanoparticles for glioma therapy is provided in new Additional file 1: Table S1.

## Nanoparticles and glioma chemotherapy

Malignant brain gliomas have a low response to anticancer therapy and a high death rate due to their aggressive and infiltrating character, as well as the presence of the BBB and overexpression of P-glycoprotein (P-gp) [40, 41]. The presence of the BBB is the most important barrier to medications entering the systemic brain-targeted delivery system. As a result, achieving the requisite concentration of chemotherapeutic medicines in the brain is difficult [42]. Furthermore, using the energy released by adenosine triphosphate (ATP) hydrolysis, the P-gp can pump chemotherapy medicines outside the cell, reducing the effective concentration of the drug in the cell and decreasing tumour cell sensitivity to chemotherapeutic treatments [43]. Chemotherapeutic medicines used to treat glioma have very limited bioavailability due to their hydrophobic and unstable nature. As a result, a substantial dose of the drug is frequently necessary, which might result in major systemic toxic side effects [44].

Glioblastoma is treated with two FDA-approved drugs: temozolomide (TMZ), a DNA alkylating agent, and bevacizumab, a humanized monoclonal antibody IgG1. Both are ineffective, therefore researchers are constantly looking for new medicines that are more successful and have fewer side effects [45, 46]. TMZ causes DNA double strand breaks, cell cycle arrest, and cell death by methylating the guanine and adenine bases [47]. TMZ, on the other hand, has a number of problems, including non-specific DNA binding, which might harm hematopoietic stem cells in patients, resulting in dose-limiting haematological toxicity [48]. Another disadvantage of TMZ is its low solubility in physiological circumstances, as well as its quick hydrolysis at slightly alkaline pH, which limits its anti-tumor activity [49]. Under physiological conditions, TMZ is spontaneously converted to its carboxylic acid counterpart, temozolomide acid (TMZA) [50].

**Fig. 2:**
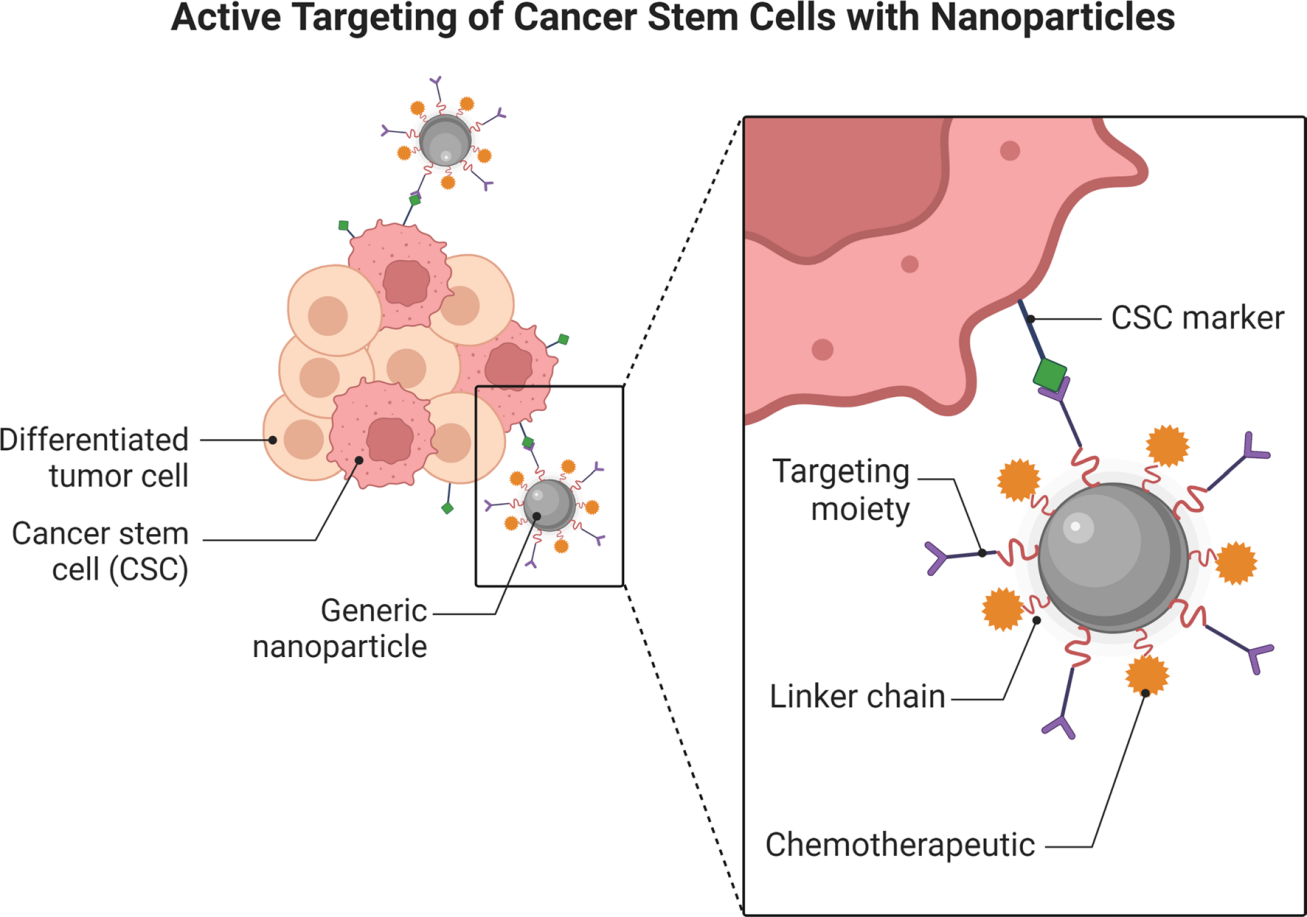
Active targeting of cancer stem cells with nanoparticles. The nanoparticles are designed to carry therapy-carrying binding chains and target moieties that target a specific CSC marker

Therapeutic drugs can be incorporated into NPs, which can then be functionalized with various ligands to allow for BBB crossing and targeting (Fig. 2). Furthermore, NPs have the benefit of shielding the therapeutic cargo from degradation and metabolism, hence improving medication stability and decreasing unwanted side effects [51, 52]. Nanocarriers made of organic and inorganic materials such as metals, silicon, carbon, and other polymers have been employed to improve the delivery of a variety of therapies. Biological materials like as proteins and lipids, on the other hand, have a better efficiency in the production of translational nanotherapeutics [19].

Glioma 261 (GL261) and brain cancer stem cells were targeted with temozolomide acid (TMZA) encapsulated into human serum albumin nanoparticles (HSA NPs). The chosen formula was discovered to have a long shelf life and serum stability, but a quick drug release pattern. After 24 h of incubation with the GL261 and BL6 glioblastoma cell lines (BL6), the optimised NPs demonstrated good cellular uptake, with 50 and 100 percent of cells positive for NP uptake, respectively. After 72 h of incubation, the chosen formula showed high cytotoxicity, with 20% cell viability at 1 mM TMZA. Finally, after intravenous delivery, the fluorescently labelled NPs displayed co-localization with the bioluminescent syngeneic BL6 intracranial tumour mouse model [53].

Nanomedicines are a promising way to deliver different therapeutic modalities to tumours in a safe and regulated manner by releasing cytotoxic payloads only in tumour tissue. Highly PEGylated hyperbranched polymers (HBPs) can be used to deliver chemotherapy in a pro-drug form to the tumour site, and this has been established in recent preclinical models with minimal adverse effects [54, 55]. It accomplishes this by avoiding extensive immune recognition thanks to the polyethylene glycol (PEG)-side-chains’ in vivo stealth properties, and then releasing the drug in the tumour environment; typically, this is accomplished by cleaving a drug linker containing an acid-cleavable hydrazone bond under tumor-specific conditions [56]. HBPs’ heightened EPR in brain tumours also helps with drug delivery, as leaky vasculature and inadequate lymphatic drainage cause nano-sized materials to accumulate within the tumour. Targeting these nanocarriers to common glioma-specific markers, such as Ephrin A2 (EphA2) [57], epidermal growth factor receptor (EGFR), and others [58], might further improve personalized specificity and accumulation. Several anticancer drugs have been delivered to the brain via nanosystems, including temozolomide [35], paclitaxel [59], docetaxel [60], cisplatin [34], doxorubicin [33], curcumin [61], and nucleic acids [66]. The most widely used to deliver these chemotherapeutics to GBM are solid lipid nanoparticles [60], polymeric nanoparticles [62], micelles [63], gold nanoparticles [64], superparamagnetic iron oxide nanoparticles [65], and nucleic acids [66].

The particular binding process between transferrin (TF) and its receptor has recently inspired several researchers. Overexpression of TF receptors in glioma cells significantly improves the ability of TF-carrying malignancy therapies to traverse the BBB [67]. As a result, the discovery of effective antiglioma medicines that contain TF is becoming more common. There are several study data on drug modification using nanotechnology among them. The nanodrug delivery system presents a new option for the effective treatment of malignant glioma due to its unique size and excellent drug binding capabilities. PEG-DSPE (polyethylene glycol distearoyl phosphatidyl ethanolamine) is a PEG derivative that comprises both hydrophilic and hydrophobic segments. As a result, PEG-DSPE is frequently used as a nanodrug carrier [68]. Glioma tests reveal that the nanodrug can successfully traverse the BBB and exert an anticancer impact. Exosomes have recently been intensively researched as a medication delivery vehicle [69]. Exosomes have been utilized to carry specific siRNAs and mediate gene knockdown in the brain because they can cross the BBB [70]. The potential of exosome-coated drug loading nanoparticles in the treatment of breast cancer has been investigated [71]. Importantly, this drug-loading approach has been proven to deliver DOX to breast cancer cells, resulting in effective apoptosis and tumour growth reduction [71]. Although DOX’s effectiveness in treating GBM is limited by its inability to penetrate the BBB, it has been demonstrated in vitro that DOX can effectively induce GBM cell death and that DOX-coated nanoparticles can penetrate a model of BBB made up of a monolayer of Madin–Darby canine kidney transfected with multidrug resistant protein 1 [69].

In chemotherapy, overcome BBB is very important, and ligand modified nanoparticles have been widely used for targeting drug delivery. However, recent studies have showed using cleavable linker for ligand modification could improve the BBB transcytosis and finally improve glioma treatment [72]. The size of nanoparticles could influence the glioma targeting delivery, so studies have developed size changeable nanoparticles to improve glioma chemotherapy and immunotherapy [73–75].

The modification of targeting ligands on nanoparticles (NPs) is expected to improve therapeutic delivery to diseased tissues. However, once in the bloodstream, NPs can immediately adsorb proteins to form the “protein corona,” which can significantly impair the targeting ligand's ability to bind to its receptor. The protein corona can make it difficult for NPs to target cells and tissues [76–78]. Salvati et al. discovered that the protein corona can protect Tf from binding to both its cell receptors and soluble Tf receptors (TfR) using transferrin (Tf)-conjugated NPs [79]. Furthermore, after formation of the protein corona, NPs functionalized with antibodies, peptides, and aptamers failed to bind specific cell types [80, 81]. Furthermore, NPs that adsorb opsonins (such as immunoglobulins, complement component, and fibrinogen) are easily cleared from the circulatory system.

## Nanoparticles and glioma radiotherapy

In 2004, the European Organization for Research and Treatment of Cancer (EORTC) 26981-22981/National Cancer Institute of Canada Clinical Trials Group (NCIC CTG) reported that adding concomitant and adjuvant MZ, an oral alkylating agent, to radiotherapy (RT) after maximal safe resection improved progression-free and overall survival for patients with GBM [82].

Two more recent trials looked at the treatment of newly diagnosed GBM patients with RT and both concurrent TMZ and bevacizumab, but found no advantage in overall survival [83, 84]. Despite the fact that radiation is regarded standard of care for the treatment of GBM, there are still numerous areas of debate and innovation.

Radiotherapy is one of the most common therapies for glioblastoma, although it frequently encounters the phenomena of radio resistance, which reduces its efficiency. Glioblastoma stem-like cells (GSCs) and dominant clones, two separate cell populations, have recently been linked to resistance [85]. It is distinguished by the activation of signalling pathways as well as DNA repair processes. Nanomedicine’s recent advancements have opened up new opportunities for radio sensitizing these cell types. In this direction, several techniques have been devised, the first of which involves encapsulating a contrast agent or manufacturing metal-based nanocarriers to concentrate the dose gradient at the target tissue level. In the second technique, the vectors' physicochemical features are employed to enclose a wide range of pharmacological compounds that work in tandem with ionizing radiation to kill malignant cells [85].

Radiation affects cell membrane permeability by causing phospholipid degradation, as well as ribosomes and mitochondria in the cytoplasm [86], although the biological effects of irradiation are mostly due to DNA lesions, which include highly lethal double-strand (ds) breaks [87]. They can be direct, as a result of interactions between particles (for example, photons for external beam radiation) and DNA molecules, or indirect, as a result of interactions with reactive oxygen species (ROS) produced by cell water radiolysis [88].

External radiation therapy (EBRT) and internal radioisotope therapy are the two types of radiotherapy (RT). Radiation beams from outside the body, such as high-energy X-rays, electron beams, or proton beams, are directly irradiated on the tumour in EBRT, causing cancer cells to die. To transfer therapeutic radioisotopes into the tumour for RT, a minimally invasive technique is used, such as direct infusion via a catheter (also called brachytherapy) [89, 90].

For whole-brain irradiation, traditional glioma radiotherapy generally uses linear accelerators, which can easily destroy normal brain tissue and alter the radiotherapy dose in the tumour area. To optimize the effect of radiotherapy, prevent tumour progression, and improve radiation damage, radiotherapy technology has increasingly evolved from whole-brain to local irradiation, with advancements and studies made when using radiosensitizers, radiation dosages, and radiation time intervals [91].

Because of the protective autophagy caused by X-Ray irradiation and tumour cells’ high ability to repair damaged DNA, GBM radio-resistance remains a significant cause of radiotherapy failure. To improve survival in non-surgical glioblastoma treatment, researchers tried to improve radiotherapy and better target pharmaceutical drugs to tumours through immunotherapy. The medical community has concluded that radiotherapy is an excellent way to improve the survival rate of cancer patients. However, it has been noted that the sensitivity to radiation varies from person to person, leaving no guarantee of treatment efficacy. Furthermore, research have revealed that the clinical use of radiation is frequently associated with severe adverse effects (such as hair loss, skin allergy, and decreased immune function [92]. Radiotherapy can't save all of the good tissues around the malignant ones. Combinations of therapeutic modalities have been proposed to overcome these constraints, and they have already shown promising outcomes with AuNPs [93, 94]. AuNPs can also target tumours by passive EPR effect and mononuclear phagocyte system (MPS) escape) and active (tumour cell targeting and stimuli-response) targeting [95]. Because GBM is a radioresistant tumour [96] and the majority of recurrences occur in the radiation field [97] radio sensitization of the tumour is an essential objective for improving the outcome in GBM patients. Phosphoinositide 3-kinases (PI3K) pathway inhibitors [98], DNA repair inhibitors [99], hyperthermia [100], aldehyde dehydrogenase inhibitors [101], and high atomic number (high-Z) metal nanoparticles (MNPs) [102] are among the radio sensitizing techniques now in research.

Although previous research has shown that silver nanoparticles (AgNPs) improve the radio sensitivity of human glioma cells in vitro, the effect of AgNPs on hypoxic glioma cells has not been studied. AgNPs had a higher ability for radio sensitization in hypoxia cells than in normoxic cells, according to the sensitization enhancement ratio (U25, C6 cells). The radio sensitization of AgNPs in hypoxic cells is mediated by the stimulation of apoptosis and increased destructive autophagy. In AgNPs-radio sensitized hypoxic cells, there is evidence of interaction between apoptosis and autophagy, with autophagy suppression resulting in lower apoptosis. These findings imply that AgNPs could be employed to treat hypoxic glioma as a highly effective nano-radiosensitizer [103]. The radio sensitivity augmentation was aided by the activation of extracellular signal-regulated kinases (ERKs) and c-Jun N-terminal kinases (JNKs). U0126 and SP600125 were used to inhibit ERK and JNK, respectively. The amount of autophagy in cells treated with AgNPs and radiation was reduced. Furthermore, SP600125 dramatically reduced the apoptosis rate of the co-treated cells. Taken together, the findings of this work will have a significant impact on AgNP biomedical applications and clinical glioma treatment [104].

Nanoparticles provide a robust dual-targeting platform for glioma radiotherapy by simultaneously eliminating tumour cells and modifying myeloid phenotypes in the central nervous system. Irradiation for glioma treatment employing a magnetic nanoparticle-based platform with cationic polymer modification increased cytotoxicity in glioma cells under radiation and improved survival in immunocompetent and aythmic glioma rats. The magnetic properties of the nanoparticles were used by myeloid derived suppressor cells (MDSC) to take up nanoparticles in the brain tumour. The anticancer benefits were linked to the death of glioma cells and the repolarization of MDSCs from an immunosuppressive to a pro-inflammatory phenotype, which improved antitumor effects and promoted radio-therapeutic effects synergistically [105].

Radio resistance is fueled by RTP cells, which selectively activate DNA damage repair and promote stemness. Continuous radiation activates the nuclear factor kappa-light-chain-enhancer of activated B cells (NF-B) signalling cascade and promotes protein 65 (p65) nuclear translocation, resulting in increased production of “yin-yang.” (YY1), a transcription factor that directly reduces miR-103a transcription, according to mechanistic studies. Under these conditions, restoring miR-103a expression repressed the basic fibroblast growth factor-X-ray repair complementing defective repair in Chinese hamster cells 3 (FGF2-XRCC3) axis and reduced radio resistance capabilities. Tf-NPs also improved radio sensitivity and significantly increased survival [106].

By successfully alkalizing lysosomes, core–shell copper selenide coated gold nanoparticles (core–shell Au@Cu2-xSe NPs) can suppress autophagy flux. They can make tumour cells produce more sequestosome-1/ubiquitin-binding protein (SQSTM1/p62) protein without affecting their mRNA levels. To impede DNA repair, Au@Cu2-xSe NPs can induce the ubiquitination of the DNA repair protein Rad51 and promote its destruction by proteasomes. Using radiation and new Au@Cu2-xSe NPs, the simultaneous inhibition of protective autophagy and DNA repair dramatically suppressed the growth of orthotopic GBM. By purposefully creating theragnostic nano-agents that concurrently suppress protective autophagy and DNA repair in tumour cells, this research presents a new perspective and paradigm for greatly improving the efficacy of radiation [107].

Because of their good biocompatibility, innate radio sensitivity, high carrying capacity of numerous medicines, and increased penetration and retention in tumour tissues, nanomaterials have been widely used to improve the efficacy of radiation [108]. The utilization of high atomic number nanoparticles (such as gold, silver, and bismuth) to augment the radiation energy deposition in cells is the subject of nanomaterial-mediated radiotherapy sensitization research. The creation of polymer nanoparticles has accelerated research into the treatment of glioma. Small molecule medications can be chemically bonded and physically coated to target glioma tissues across the blood–brain barrier, increasing the effectiveness of glioma radiotherapy [91].

Metal nanoparticles with a high Z value have a large radiation absorption capacity and can focus radiation energy on the tumour site [109]. AuNPs’ radio sensitization impact is determined by their size and surface modification [110]. Radio sensitization effects of silver, platinum, gadolinium, and other metal nanoparticles are similar to gold nanomaterials. Liu et al. discovered that AgNPs following irradiation successfully reduced cancer cell proliferation and increased cancer cell death in malignant glioma-bearing rats [111].

Radiation-induced double-strand breaks in tumour cells are the primary cause of radiotherapy resistance, and DNA has the potential to repair double-strand breaks [112]. Nanoparticles can reduce the effectiveness of radiotherapy by downregulating repair proteins such thymidylate synthase [113] or blocking the DNA damage repair signalling pathway [114]. In mice with glioblastoma in situ, a hypoxic radiosensitizer-prodrug liposome as a carrier for the DNA repair inhibitor Dbait effectively decreased the growth of glioma in situ when combined with radiotherapy [115].

Nanoparticles for internal radiation can improve tumour vascular permeability, boost EPR, and promote uptake of the next wave of nanoparticles by combining targeted nanoparticles with radioactive isotopes [116]. Nanoparticles were also commonly employed to deliver radionuclides in the treatment of glioma [117], which has been shown to be safe and feasible [118]. Allard developed a lipid nanocapsule (LNC) that encased 188Re(188Re(S3CPh)2(S2CPh)[188Re-SSS]) to generate a lipophilic complex that may be employed as a novel type of radiopharmaceutical carrier. The median survival of rats treated with 8Gy188Re-SSSLNC was greatly improved, according to the findings. Nano-radiotherapy sensitizers can not only be increased at the tumour site by increasing penetration and retention and boosting tumour tissue targeting, but they can also be used with chemotherapy, immunotherapy, and other treatments [91].

## Nanoparticles and glioma microRNA targeting, gene therapy and glioma genome editing

NPs may be a viable option for improving the efficacy and specificity of gene treatments for GBM cell death. However, the apoptotic efficiency of GBM cells is affected by the NP type and gene therapy technique used [119]. Many of the drawbacks of standard cancer therapies, such as the inability to cross the BBB and distribute throughout brain tissue, could be solved with this nanomedicine-based technique. More optimization of the SNA platform, as well as additional testing in animal models of human glioblastoma, will be required before moving into patients.

The CRISPR–CRISPR-associated protein 9 (CRISPR/Cas9) nuclease system has lately emerged as the most promising genome editing method. The Cas9 nuclease is directed to complementary sections by an engineered single guide RNA (sgRNA), which causes Cas9 to cleave the identified DNA and form double-stranded breaks (DSBs), resulting in insertions or deletions at specified target genomic loci [120]. Currently, CRISPR/Cas9 technology has emerged as one of the most promising options for treating a number of genetic illnesses, including human cancers, due to its simplicity, adaptability, and high efficiency. However, continuing development of CRISPR/Cas9 for cancer gene therapy requires the development of safe methods for delivering Cas9 and single guide RNA to tumours in an efficient and highly specific manner [121]. It is stated that liposome-templated hydrogel nanoparticles (LHNPs) with a novel core–shell nanostructure is designed for efficient code delivery of Cas9 protein and nucleic acids. When combined with minicircle DNA technology, LHNPs deliver CRISPR/Cas9 with more effectiveness in cell culture than the commercial agent Lipofectamine 2000 and can be tailored for targeted gene suppression in malignancies, particularly brain tumours. LHNPs as a versatile CRISPR/Cas9-delivery technology that may be used to study cancer biology in the lab as well as to therapeutically translate cancer gene therapy [121].

Activated oncogenes are responsible for the progression of many malignancies. Suppression of active oncogenes, which has historically been accomplished through RNA interference (RNAi) technology, may be a viable therapy option for these tumours. However, the RNAi technique has a significant flaw in that it targets mRNAs, resulting in transitory genetic regulation while leaving the oncogene’s original copy intact. Unlike RNAi, CRISPR technology causes genetic knockout at the genomic DNA level, resulting in permanent gene deletion. As a result, the administration of Cas9/sgRNA-based gene therapy allows for a long-term therapeutic benefit.

Currently, viral vectors like as adeno-associated virus are used to deliver CRISPR/Cas9 [122]. Because of their high transduction efficiency, viral methods offer a significant advantage. Unfortunately, due to safety concerns, clinical translation of viral methods has proved difficult [123]. To get around these constraints, employing nonviral vectors to deliver CRISPR/Cas9 is a viable option [124]. Several nonviral methods for delivering CRISPR/Cas9 have recently been investigated, including hydrodynamic injection [125], cell penetrating peptides [126], and synthetic nanoparticles [127]. Some of them were quite effective in delivering CRISPR/Cas9 in vivo, but none of them were able to get across the BBB.

LHNPs (liposome-templated hydrogel nanoparticles) were devised and manufactured for effective protein and nucleic acid delivery. LHNPs were able to efficiently deliver CRISPR/Cas9 for effective cancer therapy in a mouse flank tumour model by using polo-like kinase 1 (PLK1) as a model gene. LHNPs can also be produced using an autocatalytic brain tumor-targeting (ABTT) mechanism, which we recently discovered for drug delivery to brain tumors11, for targeted CRISPR/Cas9 delivery to brain tumours. The findings imply that LHNPs are capable of delivering CRISPR/Cas9 for cancer gene therapy in a targeted manner [121].

The gene-loaded LPHNs-cRGD were successfully synthesized and could protect pCas9/MGMT from enzyme degradation. LPHNs-cRGD could target GBM cells and mediate the transfection of pCas9/MGMT to downregulate the expression of MGMT, resulting in an increased sensitivity of GBM cells to TMZ. MBs-LPHNs-cRGD complexes could safely and locally increase the permeability of the BBB with FUS irradiation in vivo and facilitated the accumulation of nanoparticles at the tumor region in orthotopic tumor-bearing mice [128].

Carboxylated branching poly (-amino ester)s that can self-assemble into nanoparticles for effective intracellular delivery of a range of proteins are produced. In medium containing 10% serum, nanoparticles facilitated rapid cellular uptake, effective endosomal escape, and functional cytosolic protein release into cells in vitro. Furthermore, in various cell types, nanoparticles encapsulating CRISPR-Cas9 ribonucleoproteins (RNPs) generated high levels of gene knock-in (4%) and gene knock-out (> 75%). In mice with engineered orthotopic murine glioma tumours, a single cerebral treatment of nanoparticles delivering a low RNP dose (3.5 pmol) elicited significant gene editing. For biological research and therapeutic applications, this self-assembled polymeric nanocarrier technology provides a flexible protein delivery and gene editing platform [129].

Small interfering RNA (siRNA) nanoparticles encircled a gold centre in spherical nucleic acids (SNAs) nanoparticles. In this case, the siRNA was used to silence the oncogene Bcl2L12 in human glioma cell lines and patient-derived tumour neurospheres. The SNA nanoparticles rapidly accumulated in the brain of mice after being given systemically, especially in tumours, suggesting their potential to traverse the BBB. The siRNA loaded SNAs were subsequently given to animals with human brain tumours. Impaired tumour growth and thus enhanced survival were seen, most likely as a result of increased glioma cell death [130].

The ability of a lipopolymeric nanoparticle (LPNP) formulation to encapsulate several siRNAs for powerful and targeted anti-BTIC therapy displays a surprise high affinity for brain tumor-initiating cells (BTICs). In rat brain tumours, direct infusion of LPNP siRNAs efficiently inhibits tumour growth and provides encouraging survival advantages. This multiplexed nanomedicine platform has a lot of promise as a tailored anti-BTIC therapy platform [131].

To transport plasmids comprising base editors (BEs) and sgRNA, researchers created poly (beta-amino esters) (PBAEs) with different backbones, side chains, and end caps. Lead PBAEs were used to produce efficient transfection and base editing in HEK-293T-sEGFP and U87-MG-sEGFP reporter cell lines. In mice with xenograft glioma tumours, a single intratumor injection of PBAE/pDNA nanoparticles resulted in the robust conversion of stopped-EGFP to EGFP, demonstrating effective gene editing by ABEmax-NG. Overall, these findings showed that both in vitro and in vivo, PBAEs may effectively transport BEs for tumour gene editing [132].

To determine the effect of this microRNA on U87 cell viability, researchers used Polyhydroxy butyrate (PHB) and polyethylenimine (PEI) nanoparticles to transfect U87 cells with miR-128-encoding plasmid. PHB-co-PEI copolymerization was used to develop and produce PHB-co-PEI nanoparticles (PEI). PHB-co-PEI with a positive charge adsorbs plasmid DNA efficiently and protects it from serum nucleases. Using the MTT assay, it was discovered that PHB-co-PEI significantly reduced cytotoxicity in U87 cells when compared to PEI alone [133].

Because it has the ability to change the flawed genetic information in tumour cells, trigger cellular death, and silence the genes responsible for multidrug resistance, gene therapy has been a promising option to overcome these treatment constraints for glioma. Non-viral vectors based on lipids have been researched for delivering genes across the BBB to the glioma cell target. Furthermore, non-viral vector-based gene therapy is a promising glioma treatment due to its low immunogenicity, ease of manufacture, ability to insert ligands into particular target cells, and capability to transport larger genes [134].

Gene-editing techniques that target the PD-1/PD-L1 pathway have gotten a lot of interest lately, but the big question is how to distribute them efficiently without generating side effects in clinical trials. A nanoparticle (NP) delivery method with a low molecular weight PEI lipid shell and a PLGA core has been created to package the PD-L1 gRNA-CRISPR/Cas9 plasmid and transfect human U87 glioma cells overexpressing PD-L1. For visualizing transfection efficacy, a PD-L1 gRNA-CRISPR/Cas9 plasmid is created by introducing a single guide targeting the PD-L1 sequence into a GFP CRISPR/Cas9 plasmid. These results show that NPs constructed from a cationic branched PEI lipid shell and a PLGA core are safe and effective at delivering the CRISPR/Cas9 system to U87 cells. Using NPs as a delivery vehicle, pathological gene editing in human glioma cells with the PD-L1 GFP-CRISPR/Cas9 plasmid could give a novel immunotherapy platform to treat GBM [135].

The PEI-coated Fe_3_O_4_ nanoparticles (NPs) were employed as a vehicle for therapeutic siRNA delivery in GBM cells, with an acceptable NP/siRNA weight ratio, effectively reducing cell proliferation and migration. These could be a novel therapy option for GBM patients [136].

## Nanoparticles and immunotherapy for glioma

**Fig. 3:**
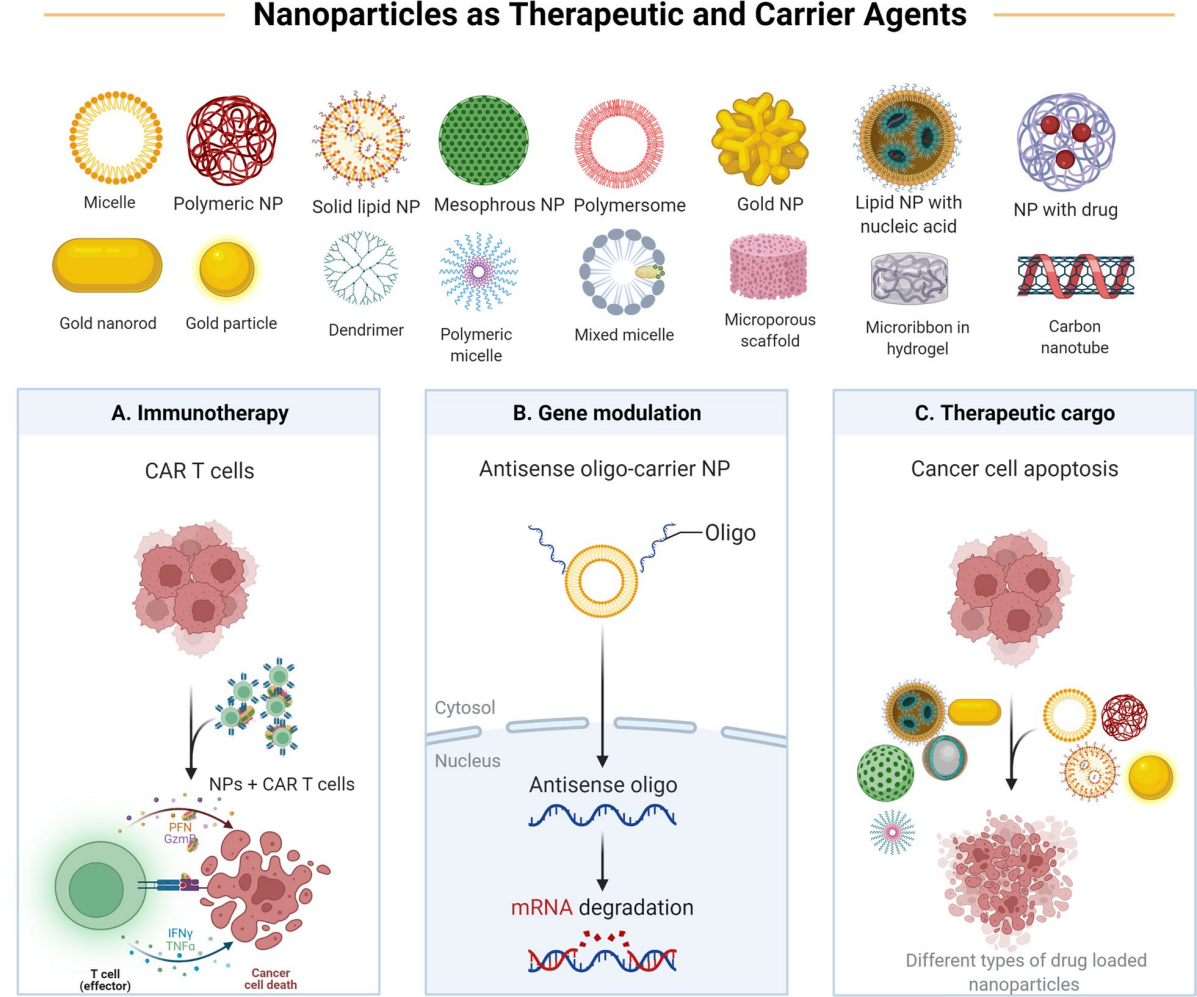
Nanoparticles as a therapeutic targets and carrier agents. Note different types of nanoparticles used to transport different therapeutic modalities, including chemotherapy, radiotherapy and immunotherapy (CAR T, mAbs, genome editing and MicroRNA)

The brain has long been thought to be immune-privileged [137]. The BBB restricts immune cell access, there is no lymphatic outflow, antigen-presenting cells are uncommon, and the major histocompatibility complex (MHC) is downregulated [137]. Some brain disorders, such as multiple sclerosis and neurodegenerative diseases, have been recognized to have active immune systems. Furthermore, when the BBB is damaged, such as by injury, immune system cells are able to infiltrate the brain [138]. The brain possesses both innate and adaptive immune systems, according to current knowledge [137]. Downregulation of MHC, increased expression of death ligand-1, and enhanced recruitment of regulatory T cells all contribute to the immunosuppressive characteristic of glioblastomas [139]. TGF, interleukin-10 (IL-10) and VEGF are immunosuppressive cytokines released by cells in the glioblastoma microenvironment [139]. Monocyte and dendritic cell activity is frequently diminished, as is the amount of invading T cells [139, 140]. Furthermore, tumor-associated macrophages, which are either immunopermissive (M1 type) or immunosuppressive (M2 type), are one of the most commonly found cells in glioblastomas (M2 type). Immunosuppressive tumor-associated macrophages have been linked to a worsening of patient outcomes [139, 141]. M1 presence in tumours is also linked to anti-tumor capabilities and the potential for tumour reduction [142]. Glioblastoma individuals have aberrant cellular immune systems throughout their bodies. The expression of PD-L1 in macrophages in the peripheral blood of patients is higher [143]. T lymphocytes are also sequestered from the circulation into the bone marrow due to sphingosine 1-phosphate receptor 1 (S1PR1) downregulation [140]. Patients with glioblastoma are typically treated with chemotherapy and radiotherapy, as well as glucocorticoids, which can alter immune system function [137]. PD-L1 expression and immunosuppressive macrophage activation have been demonstrated to be increased by radiotherapy and chemotherapy [144]. Glioblastoma’s immunosuppressive characteristic has prompted the development of a number of immunotherapeutic methods, which are described in the next section and shown schematically in Fig. 3. These includes: Chimeric Antigen Receptor T-cells (CAR T-cells), immune checkpoint modulators, immune Photothermal therapy, and vaccine-based immunotherapy.

### Nanoparticles and Chimeric Antigen Receptor (CAR) T-cells

Chimeric antigen receptors (CAR) are receptors that identify certain surface proteins and are synthesized. They are made up of single chain variable fragment (scFv) and are generated in the extracellular domain. CD3 is the intracellular component, and it contains immunoreceptor tyrosine-based activation motifs [145, 146]. In third-generation CAR, the intracellular domain has two costimulatory domains in addition to CD3, such as CD3-CD28-OX40 or CD3-CD28-41BB [145, 147]. CAR-T cells targeting the antigens Interleukin-13 receptor alpha 2 (IL13R2), human epidermal growth factor receptor 2 (HER2), and epidermal growth factor receptor variant III (EGFRvIII) have been produced in glioblastoma [146]. IL13R2 CAR-T has been used in glioblastoma treatment in a few clinical trials [146]. T-cell therapy benefits from nanoparticles. T-cells must be grown ex vivo before being processed for application. Various nanoparticles that operate as antigen-presenting portions have been proposed to improve expansion and minimize cost (Fig. 3). Polystyrene beads, liposomes, iron/dextran nanoparticles, and carbon nanotubes are among them. The most attention has been paid to Dynabeads, which are generally made of Fe3O4 in a polystyrene matrix and coated with anti-CD3 and anti-CD28 antibodies. They have consistently demonstrated T-cell expansion, are simple to handle, and have lower costs [148]. Fadel et al. developed antigen-attached carbon nanotubes in a similar way. Furthermore, IL-2 was combined with magnetite in poly(lactide-co-glycolide) nanoparticles. The nanoparticles stimulated T-cell proliferation with a 1000-fold lower use of IL-2, according to the researchers [149]. Nanoparticles could potentially be used to examine and track T-cells in real time. T-cells expressing melanoma-specific T-cell receptors were generated and tagged with gold nanoparticles by Meir et al. T-cells were subsequently injected into melanoma-bearing animals, and T-cells were tracked using CT. In contrast to non-engineered cells, the authors found that designed cells aggregated at the tumour location. The capacity to investigate the kinetics and biodistribution of T-cells is a key feature of the devised technique [150]. Nanoparticles can also be injected into the body along with T-cells. Tsao et al. created a poly(ethyleneglycol)-g-chitosan-based degradable hydrogel. At low temperatures, the gel is a liquid, and at higher degrees, it becomes a gel. The gel's goal was to encapsulate T-cells and manage their release in order to treat glioblastoma. In vitro, lymphocytes released from the gel killed glioblastoma cells, and poly(ethyleneglycol)-g-chitosan was more effective than Matrigel. Biocompatibility, degradability, and minimal immunogenicity are all advantages of poly(ethyleneglycol)-g-chitosan [151]. The use of photothermal therapy to improve T-cell therapy is another way to improve it. Chen et al. created poly(lactic-co-glycolic) acid nanoparticles that were loaded with the NIR dye indocyanine green. The nanoparticles were put into melanoma tumor-bearing mice. CAR-T against chondroitin sulphate proteoglycan-4 was also injected after that. After photothermal therapy, T-cell effectiveness rose, and the combined therapy had a therapeutic effect [152].

### Nanoparticles and immune checkpoint modulators

Tumors frequently develop a variety of defense mechanisms to prevent being eliminated by the immune system. Inhibition of T-cell response is one of the strategies. When certain antigens are exposed to T-cells, they become activated and attack tumour cells. Co-inhibitory molecules such as cytotoxic T-lymphocyte-associated antigen 4 (CTLA4) and PD-1, on the other hand, prevent the cytotoxic T-cell response [153]. As a result, drugs that target CTLA4 and PD-1 hold a lot of promise as cancer treatments. Ipilimumab, a monoclonal antibody that targets CTLA4, has already been licenced by the FDA and the European Medicines Agency (EMA) for the treatment of melanoma. Rovelizumab, another CTLA4 antibody, has been licenced for the treatment of mesothelioma [154]. The FDA has also approved the anti-PD1 antibodies nivolumab and pembrolizumab for the treatment of melanoma and non-small-cell lung cancer (NSCLC) [155]. Atezolizumab and avelumab, two anti-PD-L1 antibodies, have also been approved [154]. There are a slew of preclinical studies and clinical trials testing immune-checkpoint modulators as cancer treatments. Glioblastomas have a high level of PDL1 expression. PDL1 expression is seen in 88 percent of freshly diagnosed glioblastomas and 72 percent of recurrent glioblastomas, according to the study. The expression is likewise very low in healthy tissue around it. Furthermore, the expression is much higher than in melanoma and NSCLC (about 30% and 25%–36 percent higher, respectively). PDL1 suppresses T-cell activation and lymphocyte production of Interferons (IFNs), IL-2, and IL-10 in glioma cells [153]. Due to the high expression of PDL1 in glioblastoma, anti-PD1/PDL1 monotherapy or combined therapy has received a lot of attention [153]. Immune checkpoint modulator monotherapy appears to be ineffective in glioblastoma. In recurrent glioblastoma patients, nivolumab and pembrolizumab monotherapy did not show any benefit in terms of patient survival [156]. Many clinical trials for immune checkpoint modulators in combination with chemotherapy, radiation, bevacizumab, or other treatment modalities are now underway [156]. Many other possible modulators have also been developed, such as those targeting indoleamine 2,3-dioxygenase (IDO). Patients with solid tumours or glioblastomas are being tested in clinical trials [157]. Immune checkpoint modulators have not been shown to be useful in the treatment of glioblastoma in general. They have, on the other hand, been shown to be safe and tolerated in therapeutic settings [156].

Antigen-capturing nanoparticles may boost the efficacy of immune-checkpoint inhibitors in the treatment of glioblastoma. These are nanoparticles with a surface modification that can bind tumour antigens. Non-covalent hydrophobic–hydrophobic interactions, as well as ionic or covalent interactions, generally alter the surface [158]. Min et al. created a poly(lactic-co-glycolic) acid-based nanoparticle. They demonstrated that different surfaces had variable antigen collecting capacities. Furthermore, poly (lactic-co-glycolic) acid and 1,2-dioleoyloxy-3-(trimethylammonium) propane showed the best ability to bind tumour antigens. After that, the nanoparticles were administered into mice with melanoma who were receiving anti-PD-1 therapy. The antigens were successfully delivered to dendritic cells by the nanoparticles, with a total response rate of 20%. Overall, the study found that biodegradable and biocompatible nanoparticles can improve antigen presentation and survival rates significantly [159]. Zhang et al. created PD-1 receptor-expressing cell membrane vesicles that were also packed with an inhibitor of indoleamine 2,3-dioxygenase (IDO). After that, vesicles were introduced into animals with melanoma tumours. Combined therapy was found to be more effective than single therapy in reducing tumour growth and improving mouse survival [160].

### Nanoparticles and immune photothermal therapy

Immune photothermal therapy aims to eliminate primary cancers as well as malignancies in many places. Immune checkpoint inhibitors in combination with plasmonic gold nanostars, which are used in photothermal therapy, were created by Liu et al. [161]. Light energy is efficiently converted into heat by gold nanostars. The therapy was put to the test in vivo on bladder cancer patients and showed encouraging outcomes. In general, combination therapy outperformed anti-PD-L1 monotherapy in terms of outcomes. The mice had a 40% survival rate and established long-lasting immunity against cancer cells [162]. Peng et al. generated NLG919/IR780 micelles with a size of less than 50 nm in another investigation. NLG919 is an IDO inhibitor, and IR780 can absorb light in the near-infrared spectrum. Micelles were given to animals with malignancies in their breasts. Primary tumours were successfully destroyed, and secondary cancers were stopped from growing, thanks to a combination of photothermal therapy and immunotherapy. T-reg activity was also reduced, whereas the presence of CD8+ T-cells increased [163].

Hollow gold nanoshells were packed together with anti-PD1 peptide in poly(lactic-co-glycolic acid) nanoparticles in a work by Luo et al. Photothermal therapy with gold nanoshells was used, and immunotherapy with anti-PD-1 was applied. The nanoparticles were then put into tumor-bearing mice. The mice were exposed to near-infrared light, which allowed the nanoparticles to release peptides. Later, CpG was added to a vaccine made of poly (lactic-co-glycolic acid), and the combination therapy caused dendritic cells to mature and produce cytokines. The therapy reduced the main tumour, stopped the growth of metastases, and prevented the formation of new tumours, which were all extremely positive outcomes [164].

### Nanoparticles and vaccine-based immunotherapy

Vaccine-based immunotherapy has primarily been explored in rodent animal models and has involved a variety of techniques, including glioma cell modification, dendritic cell application, peptide-based vaccinations, and a combination approach with various treatment modalities [165]. Mutant IDH, EGFRvIII, a panel of antigens, or even personally selected antigens are the most prevalent targets in glioblastomas. In glioblastoma, the EGFR gene is increased in 40% of cases, and exon 2–7 is deleted or mutated in more than 50% of cases [165, 166]. Because the mutant form of the protein lacks a ligand-binding domain, it has constitutive activity, which promotes cancer. Several kinases, including Src family kinases, can also activate the mutant receptor [166]. The altered amino-acid sequence has been discovered to be immunogenic, and a vaccine called rindopepimut has been produced to stimulate the immune system [167]. The vaccine showed promise in early clinical trials. The subsequent phase III clinical trial, however, failed to show any improvements in overall survival [168]. Patients with recurrent glioblastomas were given either control or rindopepimut in another clinical trial called ReACT (A Study of Rindopepimut/GM-CSF in patients with relapsed EGFRvIII-Positive Glioblastoma). The vaccine outperformed the control group, with a median survival of 12 months against 8.8 months [169]. The use of nanoparticles in vaccinations can improve their efficacy. They have the ability to shield the vaccine against degradation while also increasing APC absorption. Kuai and colleagues created nanodiscs using lipids and peptides obtained from high-density lipoprotein. The surface was then coated with antigen peptides and cholesterol. The nanodiscs were more effective in priming T cells in animals with melanoma tumours, and the impact lasted longer. Nanodiscs combined with anti-PD-1 therapy produced tumour regression in 88 percent of mice with tumour models, which was significantly higher than either of them alone [170]. Alternatively, the vaccination could be in the form of mRNA. Liu et al. synthesised lipid/calcium/phosphate nanoparticles that conveyed mucin 1 (MUC1) mRNA. The vaccine was subsequently combined with anti-CTLA-4 antibody and injected into animals with triple-negative breast cancer. The combined therapy had a better response than either therapy alone [171].

Nanoparticle platforms can be engineered to deliver tumour antigens, whole tumour cells, and chemotherapeutic or phototherapeutic agents in such a way that the host’s immune system is effectively and safely triggered against tumour cells. Nanovaccines provide a one-of-a-kind platform for the delivery of personalised tumour neoantigens and adjuvants, as well as the induction of robust immune responses against aggressive tumours. Approaches based on antigens and whole tumour cells may pave the way for personalized cancer vaccination and immunotherapy. Finally, building on recent advances in nanoparticle-based cancer immunotherapy, the field should aim for clinical translation and clinical efficacy as its ultimate goal. The regulatory, analytical, and manufacturing roadblocks that need to be overcome in order to accelerate the clinical translation of nanomedicine-based cancer immunotherapy [172].

## Conclusions

Glioblastoma is the most aggressive brain tumor and current treatment is unsuccessful, resulting in low patient survival rates and poor prognosis. Nanomedicine, which would improve diagnostics, enable efficient targeting and transport of drugs across the BBB, improve drug solubility, extend bloodstream half-life, and allow regulated and sustained drug release, is an option. to resolve these difficulties. Many alternative nanomedicine formulations are being evaluated in cell lines and animal models to develop a nanomedicine combination with minimal toxicity, biocompatibility, and specificity for cancer cells. Pegylated liposomes with doxorubicin, either as monotherapy or in combination with temozolomide, are the only nanomedicines currently in clinical trials. The use of fluorescent probes as imaging agents for intraoperative MRI has the potential to improve quality of life by maximizing the surgical resection area. In addition, the personalization of these probes is possible, making it possible to adapt the diagnostic and therapeutic processes to the needs of the patients. Immunotherapy methods for the treatment of cancer, especially glioblastoma, have become popular in recent years. Treatments, on the other hand, have not yet proven to be very effective. The existence of BBBs and their immunosuppressive nature are the main reasons for their failure. Nanoparticles have been proposed as a way to improve immunotherapy in glioblastomas. They showed particularly promising results when photothermal treatment was used in conjunction with immune checkpoint inhibitors. In general, designing effective treatments for glioblastoma disease is difficult due to significant tumor heterogeneity, which means that the drug of choice may be effective for one cell type but useless for others in the tumor. Accordingly, a number of integrated techniques are being considered and are considered more effective in eradicating diseased cells. Nanoparticles used correctly in precise medicine will ultimately result in a better percentage of long-term survivors.

## Supplementary Information

**Additional file 1: Table S1.** ClinicalTrials.gov search results 08/01/2022.

## Data Availability

All data are available in the manuscript.

## Abbreviations

BBB: Blood–brain barrier
CAR T cells: Chimeric antigen receptor T-cells
EGFR: Epidermal growth factor receptor
PDGFs: Platelet-derived growth factors
TP53: Tumor protein 53 gene
CDKN2A: Cyclin-dependent kinase inhibitor 2A
PTEN: Phosphatase and tensin homolog
GBM: Glioblastoma multiforme
WHO: World Health Organization
TME: Tumour microenvironment
PN: Polymeric nanoparticles
SLN: Solid lipid nanoparticles
EPR: Increased permeability and retention
ROS: Reactive oxygen species
GLUT1: Glucose transporter 1
AuNPs: Gold nanoparticles
P-gp: P-glycoprotein
ATP: Adenosine triphosphate
FDA: Food and Drug Administration
TMZ: Temozolomide
TMZA: Temozolomide acid
HSA NPs: Human serum albumin nanoparticles
GL261: Glioma 261
BL6: BL6 glioblastoma cell lines
NPs: Nanoparticles
MDR: Multidrug resistance
ABC: ATP-binding cassette
HBPs: Highly PEGylated hyperbranched polymers
PEG: Polyethylene glycol
EphA2: Ephrin A2
PTX: Paclitaxel
CRT: Calreticulin
DAMP: Damage-associated molecular pattern
BBTB: Blood–brain tumour barrier
NPPTX: A glutathione (GSH)-responsive prodrug nanoparticle
IL-13R2: Interleukin 13 receptor 2
DOX: Doxorubicin
TF: Transferrin
PEG-DSPE: Polyethylene glycol distearoylphosphatidylethanolamine
EORTC: European Organization for Research and Treatment of Cancer
NCIC CTG: National Cancer Institute of Canada Clinical Trials Group
RT: Radiotherapy
EBRT: External radiation therapy
MPS: Mononuclear phagocyte system
PI3K: Phosphoinositide 3-kinases
MNPs: Metal nanoparticles
AgNPs: Silver nanoparticles
ERKs: Extracellular signal-regulated kinases
JNKs: C-Jun N-terminal kinases
MDSC: Myeloid derived suppressor cells
Core–shell Au@Cu2-xSe NPs: Core–shell copper selenide coated gold nanoparticles
SQSTM1/p62 protein: Sequestosome-1/ubiquitin-binding protein
NF-B: Nuclear factor kappa-light-chain-enhancer of activated B cells
YY1: Yin-yang
FGF2-XRCC3: Basic fibroblast growth factor-X-ray repair complementing defective repair in Chinese hamster cells 3
Tf-NPs: Transferrin-nanoparticles
SQSTM1/p62: Sequestosome-1/ubiquitin-binding protein
LNC: Lipid nanocapsule
CRISPR/Cas9: CRISPR–CRISPR-associated protein 9
sgRNA: Single guide RNA
DSBs: Double-stranded breaks
LHNPs: Liposome-templated hydrogel nanoparticles
RNAi: RNA interference
PLK1: Polo-like kinase 1
ABTT: Autocatalytic brain tumor-targeting
RNPs: Ribonucleoproteins
siRNA: Small interfering RNA
SNAs: Spherical nucleic acids
LPNP: Lipopolymeric nanoparticle
BTICs: Brain tumor-initiating cells
PBAEs: Poly (beta-amino esters)
PHB: Polyhydroxy butyrate
PEI: Polyethylenimine
PD-L1: Programmed death-ligand 1
PD-1: Programmed cell death protein
MHC: Major histocompatibility complex
TGF: Transforming growth factor beta
IL-10: Interleukin-10
VEGF: Vascular endothelial growth factor
M1: Macrophage 1
M2: Macrophage 2
S1PR1: Sphingosine 1-phosphate receptor 1
ScFvs: Single chain variable fragment
IL13R2: Interleukin-13 receptor alpha 2
HER2: Human epidermal growth factor receptor 2
CTLA4: Cytotoxic T-lymphocyte-associated antigen 4
EMA: European Medicines Agency
NSCLC: Non-small-cell lung cancer
IL-2: Interleukin-2
IDO: Indoleamine 2,3-dioxygenase
MUC1: Mucin 1
