# Supplementary material for: Multimodal targeting of glioma with functionalized nanoparticles
Source: Cancer Cell Int. 2022 Aug 23;22:265. doi: 10.1186/s12935-022-02687-8 (PMC9396820; doi:10.1186/s12935-022-02687-8)
Supplement: Supplementary file 1 — Additional file 1: Table S1. ClinicalTrials.gov search results 08/01/2022. [file 12935_2022_2687_MOESM1_ESM.pdf]

ClinicalTrials.gov Search Results 08/01/2022

	Title	Status	Study Results	Conditions	Interventions	Locations
1	MTX110 by Convection-Enhanced Delivery in Treating Participants With Newly-Diagnosed Diffuse Intrinsic Pontine Glioma	Completed	Has Results	•Diffuse Intrinsic Pontine Glioma	•Drug: Panobinostat Nanoparticle Formulation MTX110<br>•Drug: Convection-Enhanced Delivery (CED)	•University of California, San Francisco, San Francisco, California, United States
2	ABI-009 (Nab-rapamycin) in Recurrent High Grade Glioma and Newly Diagnosed Glioblastoma	Active, not recruiting	No Results Available	•High Grade Recurrent Glioma and Newly Diagnosed Glioblastoma	•Drug: ABI-009<br>•Drug: Bevacizumab<br>•Drug: Temozolomide<br>•Drug: Lomustine<br>•Radiation: Radiation<br>•Drug: Marizomib	•St. Joseph Heritage Healthcare, Fullerton, California, United States<br>•Hoag Memorial Hospital Presbyterian, Newport Beach, California, United States<br>•John Wayne Cancer Institute, Santa Monica, California, United States
3	CED of MTX110 Newly Diagnosed Diffuse Midline Gliomas	Recruiting	No Results Available	•Diffuse Intrinsic Pontine Glioma<br>•Diffuse Pontine and Thalamic Gliomas<br>•Diffuse Midline Glioma	•Drug: Infusate with MTX110 and gadolinium<br>•Device: Convection-Enhanced Delivery (CED)	•Columbia University Irving Medical Center, New York, New York, United States
4	A Phase I Trial of Nanoliposomal CPT-11 (NL CPT-11) in Patients With Recurrent High-Grade Gliomas	Completed	No Results Available	•Glioblastoma<br>•Gliosarcoma<br>•Anaplastic Astrocytoma<br>•Anaplastic Oligodendroglioma	•Drug: Nanoliposomal CPT-11	•University of California, San Francisco, San Francisco, California, United States
5	Assessing Dynamic Magnetic Resonance (MR) Imaging in Patients With Recurrent High Grade Glioma Receiving Chemotherapy	Terminated	No Results Available	•Brain Neoplasms	•Drug: Ferumoxytol	•Oregon Health & Science University, Portland, Oregon, United States
6	AGuIX Nanoparticles With Radiotherapy Plus Concomitant Temozolomide in the Treatment of Newly Diagnosed Glioblastoma	Recruiting	No Results Available	•Glioblastoma	•Drug: Polysiloxane Gd-Chelates based nanoparticles (AGuIX)<br>•Radiation: radiotherapy<br>•Drug: Temozolomide	•CHU de Brest, Brest, France<br>•Centre Jean Perrin, Clermont-Ferrand, France<br>•CHU de Grenoble, Grenoble, France<br>•Centre Léon Berard, Lyon, France<br>•Institut de Cancérologie de l'Ouest, Saint Herblain, France<br>•Institut de Cancérologie Strasbourg Europe, Strasbourg, France
7	NU-0129 in Treating Patients With Recurrent Glioblastoma or Gliosarcoma Undergoing Surgery	Completed	No Results Available	•Gliosarcoma<br>•Recurrent Glioblastoma	•Other: Laboratory Biomarker Analysis<br>•Other: Pharmacological Study<br>•Drug: Targeted Molecular Therapy	•Northwestern University, Chicago, Illinois, United States
8	Phase II Study of Combined Temozolomide and SGT-53 for Treatment of Recurrent Glioblastoma	Terminated	Has Results	•RECURRENT GLIOBLASTOMA	•Genetic: SGT-53<br>•Drug: Temozolomide	•MD Anderson Cancer Center, Houston, Texas, United States<br>•China Medical University Hospital, Taichung, Taiwan

	Title	Status	Study Results	Conditions	Interventions	Locations
9	9-ING-41 in Patients With Advanced Cancers	Recruiting	No Results Available	•Cancer<br>•Pancreatic Cancer<br>•Sarcoma<br>•Renal Cancer<br>•Refractory Cancer<br>•Refractory Neoplasm<br>•Refractory Non-Hodgkin Lymphoma<br>•Pancreatic Adenocarcinoma<br>•Resistant Cancer<br>•Neoplasm Metastasis<br>•and 15 more	•Drug: 9-ING-41<br>•Drug: Gemcitabine - 21 day cycle<br>•Drug: Doxorubicin.<br>•Drug: Lomustine<br>•Drug: Carboplatin.<br>•Drug: Nab paclitaxel.<br>•Drug: Paclitaxel.<br>•Drug: Gemcitabine - 28 day cycle<br>•Drug: Irinotecan	•Mayo Clinic, Phoenix, Arizona, United States<br>•UCSF Helen Diller Family Comprehensive Cancer Center, San Francisco, California, United States<br>•Miami Cancer Institute, Miami, Florida, United States<br>•Robert H. Lurie Comprehensive Cancer Center of Northwestern University, Chicago, Illinois, United States<br>•Kansas University Cancer Center, Kansas City, Kansas, United States<br>•University of Michigan, Ann Arbor, Michigan, United States<br>•Rhode Island Hospital, Providence, Rhode Island, United States<br>•Sanford Research, Sioux Falls, South Dakota, United States<br>•Vanderbilt-Ingram Cancer Center, Nashville, Tennessee, United States<br>•Fred Hutchinson Cancer Research Center, Seattle, Washington, United States<br>•and 3 more
10	A Study to Evaluate the Safety, Tolerability and Immunogenicity of EGFR(V)-EDV-Dox in Subjects With Recurrent Glioblastoma Multiforme (GBM)	Unknown status	No Results Available	•Glioblastoma<br>•Astrocytoma, Grade IV	•Drug: EGFR(V)-EDV-Dox	•John Hopkins Hospital, Baltimore, Maryland, United States<br>•Lenox Hill Hospital, Northwell Health, New York, New York, United States
